# Development of clinical recommendations to improve the care of people living with chronic pain as a long term or late effect of cancer and its treatment

**DOI:** 10.1177/20494637251389064

**Published:** 2025-10-17

**Authors:** Julie Armoogum, Alison Llewellyn, Claire Foster, Diana Harcourt, Julie Hepburn, Micheal Prior, Candida McCabe

**Affiliations:** 1 1981 University of the West of England, Bristol, UK; 2 7423 University of Southampton, Southampton, UK; 3 Public Contributor; 4 Dorothy House Hospice Care, Bradford on Avon, UK

**Keywords:** cancer pain, chronic pain, cancer related pain, cancer late effects pain, qualitative, quantitative, guidelines, recommendations

## Abstract

**Background:**

Chronic pain is a common side effect of cancer treatment and is frequently cited as a top concern and unmet need for cancer survivors. This paper outlines the development of clinical recommendations to better support people with chronic pain as a long-term or late side effect of cancer and its treatment.

**Method:**

Evidence was identified from empirical research, and new insights from cancer survivors (*n* = 19) and healthcare professionals (*n* = 135), with findings informing draft clinical recommendations. These recommendations were reviewed and refined within online stakeholder engagement events, which comprised Phase 1 researcher review (*n* = 5) and Phase 2 Expert Review Panels (four groups and two one-to-one discussions). Membership of expert panels included cancer survivors living with chronic pain after cancer, and clinical, research, and education experts (*n* = 16). Data generated from Expert Review Panels were analysed using inductive qualitative content analysis.

**Results:**

There was shared opinion among stakeholders that the recommendations would be beneficial in this setting, the recommendations reflected the evidence and the complexity of implementation was acknowledged. Validating cancer survivors’ experiences of chronic pain was seen as essential to best practice and the importance of informed patients and healthcare professionals making good decisions together was recognised.

**Conclusions:**

Resultant clinical recommendations are summarised as: **PAINS**: **P**repare and inform, **A**cknowledge and listen, **I**ncrease healthcare professional knowledge, **N**ame and diagnose, and **S**ervices and supported self-management interventions. Implementation strategies and future research are proposed.

## Introduction

Globally, the cancer rates are increasing, and in the UK, one in two people will be diagnosed with cancer in their lifetime.^1^ Whilst cancer survival rates are improving, people can experience many long-term side effects of cancer and its treatment and pain is frequently cited as a top concern and unmet need.^2,3^ Pain that persists for 12 weeks or more is regarded as chronic pain.^4^ The International Association for the Study of Pain (IASP) defines chronic cancer-related pain as chronic pain caused by the primary cancer itself or metastases or its treatment.^5^ Prevalence rates of chronic pain in cancer survivors are reported between 40% and 50%^6–8^ and it is known to impact on quality of life,^9^ physical and mental health^10^ and financial prosperity and employment.^11^ Current chronic pain guidance advocates that cancer survivors should be screened for pain and referred to specialist pain support if required.^12–14^ However, implementation of these guidelines is unclear, as cancer survivors report challenges in accessing support services to help with their pain^3,15^ and nurses, doctors, and Allied Health Professionals have reported provision of services to support those with chronic pain after cancer treatment to be limited and inconsistent.^16,17^ The European Expert Consensus Statements on Cancer Survivorship have recognised the gap in the literature to support those living with pain as a long-term and late effects of cancer and cancer treatment and advocate for more research in this area to address patients’ needs.^18^ Further, the James Lind Alliance identified chronic pain is a top 10 Living With and Beyond Cancer research priority.^19^ Therefore, clinical recommendations to improve experiences for those experiencing chronic pain are warranted. The purpose of this paper is to describe a culmination of a body of work to develop these recommendations. It uses knowledge gathered from empirical literature and new insights from adult cancer survivors and healthcare professionals, to report the experiences of cancer survivors living with chronic pain after cancer treatment in England, UK and to consider how their experiences can be improved.

**Aim:** To develop clinical recommendations to improve the care of people living with chronic pain as a long term or late effect of cancer and its treatment.

### Objectives

• To identify, review, and synthesise the literature surrounding the experience of chronic pain after cancer in adult cancer survivors.
• To understand the support needs of cancer survivors living with chronic pain after cancer treatment.
• To draft clinical recommendations and engage with stakeholders to review and refine the recommendations.
• To produce final clinical recommendations to improve the care of people living with chronic pain as a long term or late effect of cancer and its treatment

### Target population for the recommendations

These recommendations will be aimed at the healthcare workforce who support people affected by cancer including clinicians, nurses, and allied health professionals. The recommendations have the potential to be valuable for the wider healthcare workforce, educators, researchers, commissioners, and charities to inform the planning and development of services and resources to support people living with chronic pain as a long term or late effect of cancer.

## Methods

The reporting of the methods has been guided by the AGREE reporting checklist.^20^

### Rigour and development

A programme of work was designed to meet the objectives (Figure 1). Three research studies were conducted to understand the support needs of cancer survivors living with chronic pain after cancer treatment: a qualitative evidence synthesis,^21^ a qualitative interview study with cancer survivors living with chronic pain after treatment,^15^ and a quantitative survey with healthcare professionals.^16^ The key findings across the studies were drawn together and clinical recommendations to address the key findings and improve experiences were drafted. These were discussed and refined within online stakeholder engagement events, which comprised; Phase 1 researcher review (*n* = 5) and Phase 2 Expert Review Panels (four groups and two one-to-one discussions). Final clinical recommendations were produced and reported.

**Figure 1.**
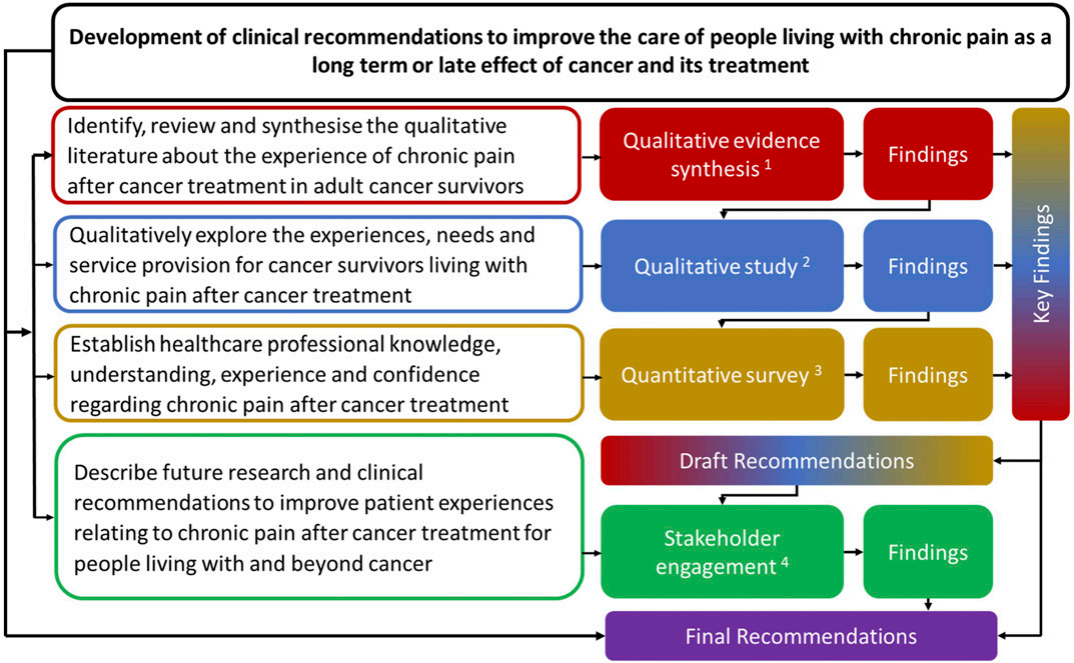
Overall programme flowchart.

#### Qualitive evidence synthesis

A full account of this study has been reported previously.^21^ The study protocol was registered with the International Prospective Register of Systematic Reviews (PROSPERO) in December 2017 (PROSPERO 2017 CRD42017082562). The original search was conducted in December 2018 and repeated in January 2023 with no additional papers identified. Data synthesis followed Thomas and Harden’s (2008) method of thematic synthesis.^22^

Findings: A paucity of studies (*n* = 4) were found, all of which focused solely on women with breast cancer. Pain sensations evoked memories of cancer diagnoses, treatment and subsequent threats to mortality, which made pain harder for an individual to manage.

#### Qualitive interviews

A detailed report of this study has been published previously.^15^ In summary, 19 cancer survivors living with chronic pain after cancer treatment participated in telephone interviews. Data were analysed with reflexive thematic analysis.^23^ National Health Service (NHS) Research Ethics and Health Regulatory Authority (HRA) approvals (19/NW/0405) and University of the West of England (UWE) Research Ethics approval (HAS.19.10.043) were obtained.

Findings: Cancer survivors (*n* = 19) identified difficult and frustrating interactions with healthcare services: survivors did not feel informed or prepared about ongoing pain, nor heard or believed. Support was hard to identify and access, and the responsibility for this was left to the survivor. Survivors experienced being bounced between services, often slipping between the gaps in provision, and they reported healthcare professionals had little knowledge about pain after cancer. However, validation of their pain by healthcare professionals was key to improving experiences.

#### Quantitative survey

Detailed methods and findings of this study have been reported.^16^ A cross sectional online survey was distributed, via professional societies, networks, and social media, to healthcare professionals including hospital and community nurses, allied health professionals, hospital doctors and General Practitioners in the UK to understand their perspectives in relation to managing chronic pain. University of the West of England (UWE) Research Ethics approval was granted (REC REF No: HAS 21.02.109). The survey consisted of four domains: (1) knowledge and understanding, (2) information and support, (3) confidence to support patients with chronic pain and (4) barriers to providing support/care to these patients. Quantitative data were analysed with descriptive statistics and free text comments were analysed using qualitative content analysis.

Findings: Healthcare professionals (*n* = 135) acknowledged the significant clinical burden of chronic pain but demonstrated mixed levels of understanding of its impact. Approximately a quarter reported they never, or rarely, talked, listened, or signposted about chronic pain after cancer.

#### Stakeholder engagement

##### Method

The aim of the stakeholder engagement was to share the key findings and draft recommendations with experts in the field, discuss how the recommendations could be met in practice and provide a qualitative description of the outcomes of the discussion. It took place in two phases.

Phase (1) Initial review of draft recommendations
Phase (2) A series of targeted engagement exercises called ‘Expert Review Panels’, to obtain a range of views, experiences and expertise to refine the clinical recommendations.

Phase 1: The research team conducted an initial review of the key findings and recommendations. A draft of the key findings and clinical recommendations was circulated by JA and an online discussion confirmed the key findings represented the evidence from across the individual studies. The importance, feasibility, and complexity of implementing the recommendations was acknowledged. As a result of the initial review, the recommendations were refined and clarified before proceeding to phase 2.

Phase 2: The composition, format, and content of the Expert Review Panels was informed by NICE and WHO guidelines,^24,25^ recommendations for qualitative fieldwork and running group interviews^26^ and prior research using consultation workshops in palliative care.

A sampling grid was created to ensure purposeful recruitment of participants with appropriate skills and experiences. Experts with experience of chronic pain and/or experience of cancer care either through clinical practice, research, education, and teaching or lived experience were subsequently recruited. These participants were identified via the UK Oncology Nursing Society (UKONS) committee members; the Pain-related Complex Cancer Late Effects Rehabilitation Service (CCLERS), Royal United Hospitals, Bath, UK; the UK Cancer Pain Nurses Group; the Aspirant Cancer Career and Education Development programme (ACCEND) steering group committee; the Radiotherapy Late Effects Special Interest Group; the Society of Radiographers; authors of relevant published literature; patient participants of previous research who had agreed to be approached for future research on this topic; and public contributors working on the research.

Participants could choose to be part of a group online meeting, run via MS Teams or have a one-to-one phone call or MS Teams meeting. MS Teams was selected as a practicable approach to enable participants to join from across the UK. All group and one-to-one meetings were facilitated by JA. JA is a university lecturer who is experienced at facilitating group meetings in an online setting. All meetings were recorded and transcribed.

Richie et al. (2014) recommend ‘enabling techniques’ to stimulate thinking and enable participants to reflect and discuss a topic more deeply. Providing information, before or during discussions can be a method to aid expression, refine views, tease out difference in views, explore priorities and look at how abstract concepts can be applied in practice. To this end, a summary of draft clinical recommendations was sent to all Expert Review Panel members via email in advance of the meetings. The summary was used as a topic guide during the Expert Review Panels. It was not an exact prescription of the order or coverage of each discussion item but steered the data collection to key questions.^26^ The discussion broadly focused on the following questions.

(a) Do these recommendations need to be refined? If so, how?
(b) To meet these recommendations, what would ‘good’ look like?
(c) How would it be measured?
(d) What could enable this?
(e) What are the challenges and barriers?

##### Analysis

Transcripts of the Expert Review Panels were analysed using qualitative content analysis. Inductive qualitative content analysis involves three phases: preparation, organisation and reporting.^27–29^ During the preparation phase each Expert Review Panel recording was re-listened to and MS Teams-generated transcripts were downloaded and read to get a sense of the whole. Recordings and transcripts were uploaded to NVivo and were organised into five content areas (Components for best practice, measurement strategies, enablers, challenges, and barriers). Both manifest (visible) and latent (underlying meaning) content were analysed. At the beginning of the organisation phase, open coding took place and categories were freely generated. After open coding, the lists of categories were grouped under higher order categories. The aim of the creation of higher order categories was not simply to bring together text that was similar or related, but rather to classify text that ‘belonged’ together to describe the phenomenon and increase understanding. Abstraction followed whereby re-organisation and re-contextualisation of categories took place as codes were compared and grouped into sub-categories and categories moved from closeness to distance from the text.

Trustworthiness of qualitative content analysis is increased when participants are appropriately knowledgeable about the subject under discussion. For this study, this was achieved by recruiting participants to the Expert Review Panel from across the sampling grid. Elo and colleagues (2014) suggest the analysis is shared with people who are familiar with the research topic to enhance credibility and ensure it is representative of the data as a whole and ‘matches reality’. In this study, a summary of development of codes, sub categories and categories were shared with the research team and public contributors to confirm relevance and representation. For the reporting phase, effort was made to report the results systematically and logically, with the use of quotations, as recommended by Elo et al. (2014).

## Findings from stakeholder engagement events

In total, 16 participants contributed to the Expert Review Panels (four group Expert Review Panels and two one-to-one discussions). Participants included six people with lived experience of chronic pain after cancer treatment and 10 stakeholders who were experts in cancer education (*n* = 2), cancer pain (*n* = 4), late effects of cancer (*n* = 3) and cancer care (*n* = 1). Nine stakeholders were registered nurses and one was a radiographer. Each group Expert Review Panel took approximately 1.5 h and the one-to-one discussions were between 20 and 45 min. Each group Expert Review Panel consisted of between two and five participants plus the facilitator and included a mix of participants with lived experience and/or clinical, education or research expertise.

Participants described the summary document and key findings and recommendations table as clear and providing a good summary.

The process of preparing and organising the data from the Expert Review Panels resulted in four categories.

(1) Validating cancer survivors’ experiences of chronic pain is essential to best practice
(2) Well informed patients and healthcare professionals making good decisions together
(3) Not seeking a perfect system, but an improved system
(4) Make the recommendations fly

The development of the categories is available in supplemental information 1.

### Validating cancer survivors’ experiences of chronic pain is essential to best practice

Across all Expert Review Panels, when discussing what ‘good’ looked like, it was clear that listening, validation and communication were reported to be at the heart of best practice. Clinicians recalled the impact it had on patients when patients were given opportunity to share their experiences and were communicated with in a compassionate, informed and supportive manner. One explained ‘*people have been brought to tears because it’s the first time somebody listens and acknowledges that they have got pain’ (Clinician, Expert Review Panel no.1)*. Patient representatives spoke of the benefit they encountered when they had felt listened to, and conversely the harm experienced when they had not, and expressed ‘*having people listen to you and hear what you say and believe what you’re saying is fundamental’ (Patient representative, Expert Review Panel no.1)*. The importance of validating and acknowledging the experience of chronic pain was regarded as key and this was seen as the cornerstone of what best practice (‘good’) would look like. It was acknowledged that the *‘artistry’* of communication was central to this *(Educator, Expert Review Panel no.3)*.

### Well informed patients and healthcare professionals making good decisions together

One participant eloquently described what ‘good’ would look like by explaining *‘good would be well informed patients and healthcare professionals that are making good decisions together (Patient representative, Expert Review Panel 1)’*. To enable this, the value of *‘shared decision making’ (Clinician, Expert Review Panel 1)* was highlighted by many and the importance of people having ‘*ownership’ (Patient representative, Expert Review Panel 1)*. The concept of ownership was considered in two ways. Firstly, by people affected by cancer being able to take ‘ownership’ of their symptoms and the factors that influenced them, such as knowledge about the risks of chronic pain and a sense of agency to seek help. A clinical diagnosis of chronic pain after cancer treatment and an explanation of the aetiology of their pain was essential to this. Secondly, ownership was mentioned in relation to healthcare professionals. It was said that *‘we [healthcare professionals] all need to take responsibility’ (Clinician, Expert Review Panel 2)* and chronic pain after cancer treatment is ‘*everyone’s business’ (Clinician, Expert Review Panel 2)*.

The central tenet for shared decision making was the importance of informed patients and healthcare professionals. Patient information, alongside reinforcement of the information, was discussed including the use of creative technologies such as podcasts, videos and interactive symptom detection websites. It was highlighted that some patients have *‘gone Googling’* to find information or support services but people cannot do that without being relatively well informed because *‘it’s only if you know the term ‘late effects’ that you can go looking for it…. So that in itself is a barrier’ (Researcher, Expert Review Panel 3)*. From a healthcare professional perspective, it was proposed that everyone involved with people’s care should know about the reality of chronic pain after cancer treatment, including those involved with providing treatments: ‘*it’s really important that [the reality of living with chronic pain after cancer treatment] gets back to the surgeon…. otherwise they don’t have an idea of the burden of the problem or what’s happening further down the line’ (Clinician, Expert Review Panel 2)*. A holistic needs assessment was considered by many as an approach to identify concerns and a way *to ‘improve person centered care, holistic care and provide higher quality cancer care’ (Researcher, one to one discussion)*. However, it was acknowledged that, before a holistic needs assessment can take place, healthcare professionals need to *‘realise that there’s a big gap and that people are–falling through - that needs to be first’ (Clinician, Expert Review Panel 2)* and without knowledgeable healthcare professionals having an awareness of the risks and impact of chronic pain after cancer treatment, the potential benefits of a holistic needs assessment are limited. Furthermore, whilst the benefits of shared decision making and assessment were appreciated, there was a sense of frustration that *‘we spend a lot of time doing holistic needs assessments and asking people about this and about that. But actually the patients I see, they want someone to sort their problems out’ (Clinician, Expert Review Panel 2)*.

### Not a perfect system, but an improved system

The complexity of the issues surrounding healthcare professional and patient education, and patient pathways was acknowledged in all Expert Review Panels. Ambitious suggestions were made for new roles and services, including a chronic cancer service to mirror acute oncology services. However, the significant challenges facing this were also identified such as issues of commissioning, education, access, and communication. At the conclusion of these discussions, many participants gave a wry smile and acknowledged it is ‘*hugely challenging’ (Patient representative, Expert Review Panel 3)*. However, it was also acknowledged that *‘every person… in the NHS, whatever, who’s awareness is moved [about chronic pain after cancer treatment] would improve the encounter in the life experience for a patient’ (Patient representative, Expert Review Panel 1)* and that any healthcare professional who *‘sits down with one patient and says the right things, then you’ve improved the system’ (Patient representative, Expert Review Panel 1)*.

The complexity and ‘messy’ nature of where these services sit was acknowledged by all Expert Review Panels. There was agreement that communication needs to improve across services, there is inconsistent provision across the UK, and referral pathways need to open up, but it was acknowledged that commissioning makes that extremely challenging. There were numerous suggestions for where a support service should be based, including hospital, community, and primary care.

### Make the recommendations fly

Many participants remarked on the importance of the findings and recommendations and mentioned how it provided evidence in an arena where there is little research. Some participants expressed their appreciation for the work and their relief and satisfaction that chronic pain after cancer treatment were receiving the attention of researchers. There was widespread enthusiasm, encouragement and advice to ensure the recommendations were taken forward.

It was acknowledged that cancer care and services are a *‘noisy’* place *(Patient representative, Expert Review Panel 3)* and *‘one of the challenges is making it a priority (Patient representative, Expert Review Panel 1)’*. The value of being *‘really ambitious’* and connecting with individuals in organisations of influence was encouraged as well as creating a sense of urgency about the work *(Clinician, Expert Review Panel 4)*. Many Expert Review Panel members mentioned the importance of aligning the work with current government policy and the way to *‘get people to pay attention to it is to look at the NHS long term plan’ (Researcher, one to one discussion)*. Also, to highlight the work to relevant and influential organisations and funders *‘to get their interest’* and show the work ‘*answers a question….that’s on the[ir] mind’ (Researcher, Expert Review Panel 4)*.

A summary of the changes to the findings and recommendations after the Expert Review Panels are included in supplemental information 2.

## Final clinical recommendations

Resultant clinical recommendations following the stakeholder engagement activities are described in Table 1 and evidence for each recommendation is described below.

**Table 1.** Clinical recommendations.

Clinical recommendation	
Prepare and inform	Prepare and inform people living with and beyond cancer about the risks of chronic pain after cancer treatment
Assess, acknowledge and listen	Assess, acknowledge and listen to experiences of living with chronic pain after cancer
Increase HCP^a^ awareness	Increase healthcare professional knowledge about the risks, impact and management of chronic pain after cancer treatment
Name and diagnose	Name and diagnose chronic pain after cancer treatment to educate, inform and validate experiences
Services and supported self-management interventions	Services and supported self-management interventions are required to provide support and rehabilitation for people living with and beyond cancer who experience chronic pain

^a^Health care professional.

### Prepare and inform people living with and beyond cancer about the risks of chronic pain after cancer treatment

People living with and beyond cancer are not prepared for chronic pain after cancer treatment.^15^ Cancer survivors have unmet informational needs regarding treatment side effects,^15,30,31^ leaving them with feelings of unpreparedness and isolation^32^ and a lack of information can exacerbate side effects in cancer survivorship.^15,30,33^

Cancer survivors report not being informed of risks of chronic pain at cancer diagnosis and during treatment and this can make their pain harder to manage.^15,21,33–36^

### Acknowledge and listen to experiences of living with chronic pain after cancer

People living with and beyond cancer do not feel listened to, heard or believed when talking to healthcare professionals about their chronic pain after cancer treatment. Cancer survivors can feel dismissed and invalidated when healthcare professionals do not listen, hear or believe them.^15,34,35^ This can prevent cancer survivors from discussing their discomfort with their healthcare professionals because of fear or concern of not being believed or being labelled as a hypochondriac, manipulating, or demanding of opioid therapy.^37^

### Increase healthcare professional knowledge about the risks, impact and management of chronic pain after cancer treatment

Healthcare professionals report a lack knowledge, understanding and confidence about chronic pain after cancer treatment.^16^ Healthcare professionals play an important role in the assessment, identification and management of pain in cancer survivors but healthcare but do not always ask about pain during cancer follow up.^16,38^ Healthcare professionals can underestimate prevalence, severity and impact of pain and pain related problems in cancer survivors.^16,39,40^ This can hinder follow up care for cancer survivors.^40^ Many authors advocate for healthcare professionals to have more education about cancer late effects and chronic pain^16,41–46^ and recognise self-perceived gaps in knowledge, skills and confidence can serve as a barrier to healthcare professionals addressing needs about long term effects of cancer with patients.^16,47^

### Name and diagnose chronic pain after cancer treatment to educate, inform, and validate experiences

Cancer survivors are significantly less likely to receive a diagnosis of cancer-related pain compared to those on active cancer treatment^48^ yet cancer survivors who receive a formal diagnosis and explanation for their chronic pain after cancer treatment feel understood and validated by healthcare professionals and better able to manage their pain^15^ Being formally diagnosed with cancer-related pain can make cancer survivors are more likely to be satisfied with their pain management treatments if they feel their healthcare professional understands the impact of their pain on their life, and is making every effort to find the best pain treatment for them.^48^ A diagnosis can provide understanding and comfort to the person living with chronic pain. Having a meaningful and acceptable explanation for chronic pain can give a person a sense of control and make the pain less threatening.^49^ The International Association for the Study of Pain (IASP) states that an accurate diagnosis and classification of pain can lead to important benefits to patients, including referral to tailored treatments, triggering support to promote patient self-management, and more specialist referrals for some patients with complex pain needs.^4^

### Services and supported self-management interventions are required to provide support and rehabilitation for people living with and beyond cancer who experience chronic pain

Living with chronic pain after cancer treatment is hard. It affects physical, psychological, social, emotional, financial, and social wellbeing.^9,10,15,21^ Yet cancer survivors have difficulty accessing support and obtaining help for their chronic pain after cancer treatment.^21,34,35,39^ Challenges in accessing support can start soon after cancer treatment has finished. Cancer survivors report they can feel abandoned by acute cancer services at the end of treatment and dismissed by pain services, particularly if pain management interventions had been seemingly ineffective.^15^ Healthcare professionals state there is a lack of available support services to refer patients to^16^ and there is inconsistent provision of support and rehabilitation services across the UK.^16,17^ This presents a challenge as people living with chronic pain after cancer treatment may not be able to receive a successful referral for support or rehabilitation services, be that generic or specialist chronic pain support. Composition of support and rehabilitation services needs consideration. Multidisciplinary team working is regarded as essential for chronic pain rehabilitation in cancer^50^ yet Galligan et al. (2022) found that only just over half (52.4%, *n* = 33) of services that support people living with cancer-related pain in the UK offered people a multi-disciplinary pain assessment.

Worldwide, there are ongoing challenges between hospital-based care and community care services with regards to communication and who is responsible for care and when. In a review of 97 articles from USA, Canada, Australia, the EU, and UK on primary care led cancer survivorship care, interdisciplinary communication was highlighted as the largest barrier from cancer specialists’ perspectives and the second largest barrier from primary care providers’ perspective.^51^ Poor communication and unclear roles between hospital and community services could result in reduced referral to chronic pain support.

## Implementation strategies for clinical recommendations

• Develop and promote educational resources for healthcare professionals

Evidence-based, multidisciplinary educational resources should be developed to increase healthcare professionals’ knowledge and understanding of chronic pain as a long term and late effect of cancer. Resources should be co-designed with clinical, research and education experts; people with lived experience of chronic pain after cancer; plus partners such as charities and commissioners. Types of resources could include videos, podcasts, e-learning, and booklets. A critical realist approach should be considered to understand how the resources could be helpful, in which context, and for whom.^52^ This could provide insight when preparing to scale up the resources to the wider healthcare workforce.

In addition, healthcare professionals should have access to high quality Continued Practice Development (CPD) courses to explore and develop their own learning and practice and enhance their confidence of late effects of cancer, including chronic pain. Any resource, educational intervention or CPD course should be evaluated to identify impacts on practice.

• Co-design patient information resources about chronic pain as a long-term and late effect of cancer

Co-design methodologies with patients, healthcare professionals and digital literacy teams, should be applied to develop patient information resources, including webinars, podcasts and leaflets to raise patients’ awareness and understanding of chronic pain after cancer treatment. To increase accessibility, resources should be available in multiple formats including online, print, and audio. These should be promoted via national charities, clinical practice, cancer organisations, and local patient events and networks. Furthermore, awareness of support services should be increased through promotion of directories of support services, such as those offered by the Pelvic Radiation Disease Association (https://www.prda.org.uk/late-effects-services/) and British Pain Society.

• Increase cancer education in pre-registration healthcare programmes

Pre-registration healthcare programmes should include cancer education to adequately prepare healthcare students to care for people affected by cancer.^53,54^ In the UK, the ACCEND Career Pathway, Core Cancer Capabilities, and Education Framework^55^ outlines that cancer education should be embedded into pre-registration healthcare programmes. Accordingly, national working groups, including representatives from the Council of Deans of Health (CODH), Higher Educational Institutions (HEI), NHS England, Northern Ireland, Wales, and Scotland and clinical practice should be formed to develop national pre-registration guidelines for clinical practice and Higher Education Institutions (HEIs) and thereby increase cancer education in pre-registration curricula.

• Identify current care pathways for cancer survivors living with chronic pain

UK core standards and guidance has recommended that cancer survivors living with chronic pain have access to specialised chronic pain services^13^ yet cancer survivors are reporting unclear and limited pathways for support.^15^ It is important to establish current care pathways to determine if these are in line with current recommendations and highlight good practice and areas for improvement.

• Co-create supported self-management interventions with stakeholders to support people with chronic pain after cancer treatment

There exists a clear need to develop supported self-management interventions for people living with chronic pain after cancer. Guided by the MRC framework for developing and evaluating complex interventions,^56^ core elements of intervention development for supported self-management for people experiencing chronic pain after cancer treatment should be explored. There should be extensive stakeholder engagement including patient and public involvement activities to enhance understanding of how supported self-management interventions for people living with chronic pain after cancer could work, for whom and in what circumstances. Public and patient activity should be mindful to include engagement with under-served communities. Key uncertainties related to the intervention development should be identified and acknowledged and considerations should be given to how these could impact on the interventions and their delivery. The interventions themselves should be refined and co-designed with stakeholder input including patient and public involvement.

## Discussion

This paper has described the development of clinical recommendations to improve the experiences of those living with chronic pain as a long term or late effect of cancer and its treatment. An implementation strategy has also been provided. Together, these provide guidance for healthcare professionals, service managers, researchers and commissioners, however, it is acknowledged that there are some inherent challenges to implementing these recommendations and strategies in practice.

Preparing and informing people living with and beyond cancer about the risks of chronic pain after cancer treatment can be complex and there is little agreement about how, when and by whom information should be given. There are competing priorities at the pre-cancer treatment stage and the perceived vast myriad of potential long term side effects can make it challenging to prioritise which ones to discuss with patients.^47^ Some patients can be overwhelmed by the amount of information they receive and can find it hard to absorb everything that is being said, especially close to cancer diagnosis.^57^ Some suggest these discussions should be postponed until patients are more receptive to this information, emphasizing the end of treatment as a prime opportunity to provide anticipatory guidance and set realistic expectations with respect to long-term effects.^47^ However, there is a risk that information will be missed or forgotten in practice, with everyone thinking that someone else is providing the information and support. Thus, whilst there is agreement that patients should be informed about the risks of chronic pain after cancer treatment, exactly when and by whom continues to be unclear.

Acknowledging and listening to people describe their experience of living with chronic pain after cancer sounds straightforward. There is shared understanding among healthcare professionals, researchers, and policy makers that listening to patients is essential and that how people are communicated with impacts on their care experience. Communication skills training in cancer care has been advocated for decades^58^ and is known to increase levels of healthcare professional empathy.^59^ However, poor communication and lack of empathy from healthcare professionals continues to perpetuate unmet need in people living with and beyond cancer.^60^ It is unlikely that healthcare professionals would advocate *not* listening to people living with and beyond cancer talk about their concerns, so it is important to consider the barriers that are preventing them from doing so. Lack of time and skill is often cited as a barrier to effectively communicating with patients.^61,62^ However, attributing poor communication solely to time restraints or lack of communication skills ignores an important nuance highlighted by the findings in this paper, that is, that participants do not feel *believed* when they speak about their chronic pain after cancer treatment. Some healthcare professionals can see cancer survivors who experience pain as ‘difficult’ or ‘complainers’, with a tendency to exaggerate their pain^40^ and cancer survivors can fear being considered a hypochondriac or manipulating and demanding.^37^ This level of judgement and prejudice will prevent healthcare professionals from truly listening and believing what cancer survivors are saying about their pain. It is therefore important that healthcare professionals are reminded of the importance of listening to people living with and beyond cancer and acknowledging their pain. For many people living with chronic pain after cancer, this listening can, in itself, be the therapeutic intervention that helps them feel supported.

Increasing healthcare professional knowledge and understanding is key to helping them appropriately assess for chronic pain in cancer survivors. Specialist cancer education can benefit healthcare students and the general and specialist healthcare workforce.^53,63–66^ However, the long term impact of cancer education is rarely reported.^67^ Furthermore, whilst Chan and colleagues’ (2022) systematic review found there are advantages to cancer survivorship education for healthcare professionals, such as increased confidence, knowledge, and behaviour change, they also explained that many papers in the review had methodological bias and weakness.^63^ Thus, whilst it is essential that educational resources are developed to address the healthcare professional knowledge gap relating to chronic pain after cancer treatment, these resources must have appropriate pedagogical underpinnings. Their development and evaluation should be informed by the relevant cancer practice and education guidelines, and teaching and learning theory and frameworks.

Naming and diagnosing chronic pain after cancer treatment can educate, inform and validate experiences for cancer survivors, however, it is recognised that it can be difficult to diagnose and determine if the pain is related to cancer or something else.^16^ Cancer survivors may have existing or increased co-morbidities and cancer recurrence needs to be excluded.^2^ These problems can result in either a lack of diagnosis, or a protracted route to diagnosis, which can frustrate both cancer survivors and healthcare professionals. To aid diagnosis, clinical guidelines and advice are being developed, however, this needs to be in conjunction with an increase in knowledge and understanding from healthcare professionals.

Accessing services for support is essential for high quality care. For all cancer survivors, there has been much improvement in cancer survivorship care in recent years, including the introduction of personalised stratified follow up pathways for cancer survivors in the UK.^68–70^ There are many models for how support can be delivered in the months and years following cancer treatment including supportive self-management, patient-initiated follow up, nurse led follow up, oncologist led follow up, follow up by general practitioners, shared care between oncology providers and primary care providers, long term and late effects clinics, and comprehensive multidisciplinary rehabilitation.^68,71^ It is known that appropriate support can reduce health crises, enhance confidence to manage and improve mental health, quality of life and other health outcomes in cancer survivors.^71^ There is also recognition by healthcare professionals that alternative models of care, such as patient initiated follow up, could result in moving from the current paternalist system to one that empowers cancer survivors to have more control.^72^ Cancer survivors need to feel they are being taken seriously and have appropriate and timely responses from clinicians.^73^

Supported self-management and patient education interventions are required to support those living with chronic pain after cancer treatment. Self-management support can reduce symptom severity in fatigue, pain, anxiety, and give rise to improvements in self efficacy.^74–78^ Interventions for supported self-management of pain in cancer survivors can be effective.^79^ However, support for self-management is not consistent and there needs to be a cultural shift to embrace these partnerships.^71,74^

## Limitations

This paper describes the development of clinical recommendations as opposed to clinical guidelines. Clinical recommendations provide suggestions to inform care whereas clinical guidelines are statements intended to optimize patient care, include an assessment of the benefits and harms of alternative care options and are developed following a recognised clinical guideline methodology.^80^ The recommendations described in this paper have been generated from empirical evidence and a series of stakeholder events, therefore, they provide useful recommendations to inform practice, research and policy, but they do not hold the status of formal clinical guidelines. The stakeholder groups used to refine the recommendations consisted mainly of nurses. To reduce potential for bias, more extensive stakeholder engagement could have been utilised, for example, to include wider representation from professional groups involved in chronic pain management including medics, primary care physicians, physiotherapists, psychologists, pharmacists and occupational therapists and the wider multidisciplinary team. Further consensus methodology could have been adopted to identify areas of prioritisation for implementing the recommendations and the recommendations could have been endorsed by a recognised professional body. Whilst the current study highlighted the importance of education, further research is needed to co-design, co-produce and evaluate patient information, healthcare professional educational resources and self-management interventions to support people with chronic pain after cancer treatment.

## Conclusions

Living in chronic pain causes cancer survivors physical, psychological, social, and economic distress and discomfort. This impacts individuals and their families, health service providers and wider society. It is essential we better support people living with chronic pain after cancer treatment to enable them to receive timely information, support, and rehabilitation to ensure they can live as well as possible. This paper has proposed clinical recommendations to improve the experiences of those living with chronic pain as a long term or late effect of cancer.

## Supplemental Material

Supplemental Material - Development of clinical recommendations to improve the care of people living with chronic pain as a long term or late effect of cancer and its treatment Supplemental Material for Development of clinical recommendations to improve the care of people living with chronic pain as a long term or late effect of cancer and its treatment by Julie Armoogum, Alison Llewellyn, Claire Foster, Diana Harcourt, Julie Hepburn, Micheal Prior, and Candida McCabe in British Journal of Pain

## Data Availability

Due to the sensitive nature of the questions asked in this study, respondents were assured raw data would remain confidential and would not be shared.*
