# Supplementary material for: Development of clinical recommendations to improve the care of people living with chronic pain as a long term or late effect of cancer and its treatment
Source: Br J Pain. 2025 Oct 17;20(3):160–73. doi: 10.1177/20494637251389064 (PMC12534838; doi:10.1177/20494637251389064)
Supplement: Supplementary file 1 [file sj-pdf-1-bjp-10.1177_20494637251389064.pdf]

Supplementary information 2: Codes, sub-categories and categories of analysis arising from expert review panels

Codes	Sub-categories	Categories
Importance of listening	Listening and validation are at the heart of best practice	Validating cancer survivors' experiences of chronic pain are essential to best practice
Validating pain experiences		
The way healthcare professionals communicate	Communication is paramount	
Holistic needs assessment	Shared decision making	Well informed patients and healthcare professionals making good decisions together
Patients having ownership of their symptoms		
Patients having sense of agency over care and pathway		
Link role in primary care		
Tailor information to stage of cancer pathway	Introduce and reinforce information over time using different ways to educate	
Reinforce risks of chronic pain over time		
Use of multimedia patient information	Use technology to create and develop patient and healthcare professionals' information and education	
Develop accessible learning for healthcare professionals		
Be realistic about your responsibilities as researcher	You have highlighted issue, future work to implement change and evaluate	Not a perfect system, but an improved system
How much can you change the world?		
Move the dial bit by bit	One encounter can make a difference	
improve the encounter for a patient		
Role of national education strategies	Clearer pathways for education and career progression	
New advanced practice roles emerging		
Connect to policy	Align recommendations to policy and priorities	Make the recommendations fly
Align with funders' priorities		
Approach funders	Be ambitious and brave with scope of recommendations	
Highlight transferability of findings		
Components of quality<br>What happens if they are not met	Assess against quality indicators<br>Create a sense of urgency	

Supplementary information 2: Summary of changes to findings and recommendations after the Expert Review Panels.

Draft key finding and recommendations	Final key finding and recommendations
Key finding: Chronic pain after cancer treatment is not discussed with people living with and beyond cancer	Key finding: People living with and beyond cancer do not feel heard or believed when talking to healthcare professionals about their chronic pain after cancer treatment
Key finding: Living with chronic pain after cancer treatment affects physical, psychological, social, emotional, financial, and social wellbeing	Key finding: Living with chronic pain after cancer treatment affects physical, psychological, social, emotional, financial, and social wellbeing yet cancer survivors have difficulty accessing support for their chronic pain after cancer treatment
Recommendation: Raise healthcare professional awareness, knowledge and understanding of chronic pain after cancer treatment and increase confidence to acknowledge and address it	Recommendation: People living with and beyond cancer who experience chronic pain after cancer treatment should have their concerns listened to, acknowledged and addressed
Recommendation: Raise awareness of chronic pain after cancer treatment amongst healthcare staff	Recommendation: Raise awareness, knowledge and understanding of chronic pain after cancer treatment among the healthcare workforce to increase their confidence to listen, acknowledge and address it with people living with and beyond cancer
Recommendation: Identify pathways for support and communicate pathways with healthcare professionals and people living with and beyond cancer	Amalgamated with the recommendation: 'People living with and beyond cancer should have access to rehabilitation and support services'
